# Thermal Limitations in Ultrafast Laser Direct Writings in Dielectric Solids

**DOI:** 10.3390/mi16090970

**Published:** 2025-08-22

**Authors:** Bertrand Poumellec, Ruyue Que

**Affiliations:** Institut de Chimie Moléculaire et des Matériaux d’Orsay, CNRS, Université Paris-Saclay, 91405 Orsay, France; queruyue@hotmail.com

**Keywords:** temperature distribution, femtosecond pulsed laser, interaction laser–dielectric solid, ultrafast laser direct writing (ULDW)

## Abstract

In the context of an ultrafast laser interacting with solids, temperature plays a special role in the transformation processes. Some of these processes can be thermally activated, while others can be either solely driven or constrained by temperature—such as refractive index change (fictive temperature), nanopore erasure, micro-bubble formation, and phase transition-like crystallization. The objective of this paper is to use a recently developed analytic approximation to understand the limitations imposed by the spatial temperature distribution and its evolution over the writing time, based on the key laser parameter combinations, and subsequently determine the boundary conditions of these parameters.

## 1. Introduction

### Where and When Does the Temperature Play a Role in the Processes?

The femtosecond (fs) pulsed laser has been recently shown to be an efficient tool for inducing glass modifications that result in significant refractive index changes, or the introduction of non-linear properties. In industrial applications, there is a strong need to write as fast as possible. However, since the searched properties usually depend on the pulse density (see for instance [1,2,3])—which, like the deposited energy density, decreases as the beam-scanning speed increases—the pulse energy (*Ep*) and/or the repetition rate (*RR*) are often increased to compensate for this. Performing this action, however, leads to an increase in local temperature, which can ultimately destroy the desired modifications. On the contrary, in some cases, a sufficiently high temperature is required to activate certain modifications, such as laser-induced density changes. There are thus thermal limitations that must be carefully evaluated, and they have not been studied clearly, taking into account the temperature oscillations produced by the pulsed laser irradiation and the thermal accumulation. The purpose of the present paper is to clarify this aspect. Note that all parameters and variables used in this paper are listed in Table A1 in the Appendix A.

**Figure A2:**
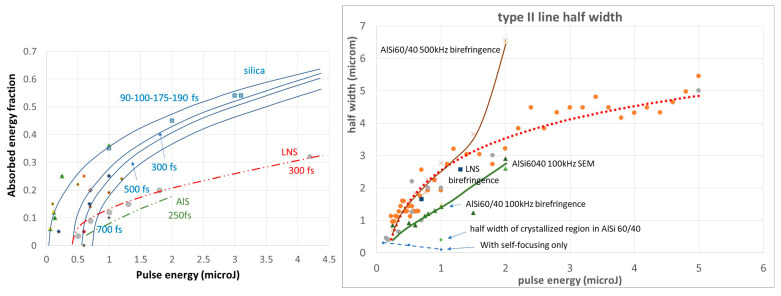
Critical parameters *A* (the absorbed energy) and *w* (the effective beam radius) for the estimation of the temperature for several glasses. Graphs are extracted from [41] and completed with line widths from our optical birefringence, SEM/TEM observations.

In the ultrafast laser–matter interaction process, energy from a laser pulse with an extremely short duration (10^−11^–10^−14^ s) is partially deposited into a small focal volume of transparent dielectric solids. This intense laser pulse, with high irradiance (>10^13^ Wcm^−2^), initiates a series of complex dynamic processes within an ultrashort timescale, including multiphoton ionization, tunneling ionization, inverse bremsstrahlung absorption, and avalanche ionization [4]. These interactions generate high-density electron excitations in the conduction band and above, creating a quasi-free electron plasma. These excitations weaken the chemical bonds, leading to rapid expansion in the focal zone and resulting in structural modifications. Subsequently, the electron energy dissipates through electron–phonon interactions [5,6], causing a rise in the material temperature. However, as discussed in [7] (based on work from [8] and proved in [9,10]), the light energy is not deposited uniformly. Light tends to concentrate around structural inhomogeneities in the glass within the incident beam area, and it also undergoes scattering with high intensity, leading to a considerable broadening of the irradiated area where the light is also absorbed (see the Appendix C, Figure A2). These inhomogeneities, acting as energy concentrators, form hot spots that lead to nanoplasma generation and eventually nanocavitation. Pending bonds and point defects with reduced bandgap can be produced after only a few pulses [11,12,13]. According to the proposed “memory effects” involved in the non-linear ionization mechanism, these defects give rise to new inhomogeneities with each successive pulse, enhancing multiple scattering. The scattering becomes so efficient that the same process can occur beyond the focal area and expand progressively.

At low incident pulse intensity (<10^12^ W/cm^2^ for 0.1 NA in silica), almost no stable structural nanomodification occurs. The energy is dissipated first through electron–phonon coupling (within a few ps), and then through thermal diffusion over the thermal diffusion timescale (corresponding to 65% of out-diffusion from the irradiated area of the deposited energy, typically a few μs in silicate). This process leads to the thermal treatment of the glass, affecting both the irradiated area and its surroundings. Such thermal effects may change the medium-range order of the glass structure, leading to fictive temperature change [14]. At the same time, the shock wave generated by rapid thermal expansion and electronic excitation leads to change in fictive pressure [15]. This pressure is determined by the cooling time, which in turn depends on the evolving spatial thermal curve [16]. As a result, this mechanism may provide an additional contribution to the refractive index change. All these effects lead to an isotropic refractive index change, commonly referred to type I modification [17,18,19,20,21]. Laser-induced crystallization in many multicomponent glasses can also produce similar isotropic changes. Moreover, due to non-linear absorption, such writing can be performed in the bulk of the material, typically a few hundred nm below the surface [22]. Notably, it is not necessary to use fs lasers to induce such processes. Isotropic crystallization has also been demonstrated using a YAG laser [23,24] and other sources [25].

At higher incident pulse intensities (>10^13^ W/cm^2^), spherical nanopores (a few nm in diameter) can form as result of high excited electron density nanoplasma generation, triggered by the concentration of light energy at pre-existing or laser-induced inhomogeneities within the glass. Under linear polarization, these nanopores rapidly become oblate after only a few pulses, due to local field enhancement, as described in [26]. In contrast, circular polarization preserves the symmetry of the nanoplasma hot spots, resulting in spherical nanopores, as observed in the so-called type X regime [27], with exhibits low optical scattering and low birefringence [28]. As the number of pulses increases, the nanopores begin to self-organize under the combined influence of the incident light polarization, multiple light scattering, and the viscosity of the glass [29]. This process leads to what is referred to as Type IIp modification [30] where ‘p’ denotes porosity to distinguish it from type IIc, where ‘c’ stands for crystallization, typically involving partial crystallization in multiple component glasses [17,31,32]. Type IIp structures exhibit strong form birefringence due to the aligned nanoporous architecture [33,34]. It is also likely that electric charge distribution plays a role in this structuring process [3,35].

Regardless of the specific type II mechanism, there exist limitations on the choice of laser parameters—mainly *Ep* and *RR*—due to the thermal stability of either the induced modification or its spatial organization. In nanopores-based type II modifications, stability is constrained by the viscosity of the glass [36], which decreases significantly when the laser-induced temperature increases. While their formation is also likely viscosity-dependent (and thus temperature-dependent), it occurs directly under the laser beam, where additional forces—especially those involved in the nanocavitation process—play a dominant role. In contrast, crystal-based type II modifications involve a more complex temperature dependence. Recent studies have shown that nanogratings are formed through a light-induced chemical separation process, involving element migration driven by thermal and/or electric potential gradients. This chemical rearrangement is then stabilized by subsequent crystallization, a thermally activated process. In this case, the thermal limitation lies in preventing bulk crystallization [31], which would destroy the anisotropic nanostructure and thus eliminate the desired form birefringence.

As the laser intensity increases further—approaching the 10^21^ W/m^2^—nanovoids can be generated through Coulomb explosion [37], a mechanism that is independent of material inhomogeneities. The thermal limitations in this regime are fundamentally similar to those for nanopores formation [38], except that the large void size (approximately ten times greater) leads to enhanced thermal stability (see Equation (1)).

More generally, if the stability of a given modification follows the behavior described by an activation energy distribution—as outlined in the VAREPA framework [39]—then the corresponding writing limitations can be directly inferred.

To explore these thermal limitations in practical contexts, we have conducted numerical simulations for several representative cases mentioned above, with silica as a model material. For this purpose, we compare the thermal stability of each considered modification to the evolution of material temperature during the writing process. The temperature evolution is evaluated using the simplified thermal model described in [40].

## 2. The Treatment Curve in Scanning Pulsed Laser Mode

We aim to estimate the material temperature a few nanoseconds after the complex processes described in the previous section—specifically, after the light energy deposition and local thermalization via electron–phonon coupling, but before the beginning of thermal diffusion. The key feature of the fs pulsed lasers is that, at any given distance from the focal center, the temperature oscillates between a maximum $T_{max}$ and a minimum $T_{min}$ within each pulse period. These temperatures depend on both the pulse energy (*Ep*) and the pulse repetition rate (*RR*). The main parameters of this problem is the ratio between the diffusion time and the pulse period. The thermal diffusion time is given by: $\tau_{D}=\frac{{w(E_{p},RR)}^{2}}{4D_{th}}$, where $\;w\left(E_{p},RR\right)\;$ is the effective beam waist radius at 1/e intensity (see Appendix C, Figure A2), and $D_{th}$ is the thermal diffusivity of the material. The pulse period is $\tau_{RR}=\frac{1}{RR}$. Note that the effective beam width (w) has been defined in [41] as the width of the form birefringent written lines.

When $\tau_{RR}\gg \tau_{D}$, the temperature around the focus center has sufficient time to return to room temperature before the arrival of the next pulse—no significant heat accumulation occurs. On the contrary, when $\tau_{RR}<\tau_{D}$ heat does not completely dissipate between pulses, it leads to cumulative heating and an increase in both $T_{max}$ and $T_{min}$ over time. This behavior can be characterized by the dimensionless ratio $R_{\tau }=\frac{\tau_{RR}}{\tau_{D}}=\frac{4D_{th}}{RR\cdot {w(E_{p,RR})}^{2}}$. The system approaches a quasi-steady state after a certain number of pulses (denoted $N_{ss}$), where the temperature oscillations stabilize. The time to reach this steady state is a fraction of ms in silica, regardless of *RR* (see Appendix B, Equation (A1)). All temperature-related quantities and oscillation behaviors were computed in [40] using a simplified model based on a spherical Gaussian focus. This model is not meant to be quantitatively exact, but rather to provide physical insights. Notably, the correct criterion for assessing heat accumulation is not simply the repetition rate, but rather the parameter $R_{\tau }$, which incorporates both the laser parameters and the material’s thermal properties. From the analysis in [40], we find the following:

- When $R_{\tau }$ is large (ca. ≳ 7), heat accumulation is negligible.
- When $R_{\tau }≲1$, cumulative heating becomes significant, and $T_{min}$ cannot be neglected. The temperature oscillations are relatively smaller.

**Figure 1:**
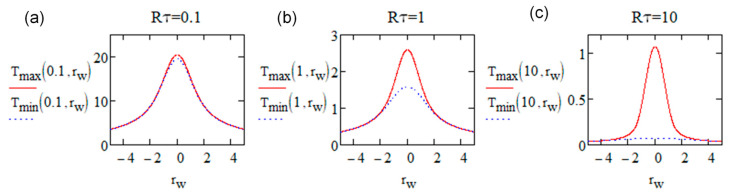
Spatial distribution of dimensionless *T_min_* (blue dash, by Equation (A3)) and *T_max_* (red, by Equation (A2)) according to the relative radius *r_w_* when (**a**) $R_{\tau }=0.1$, (**b**) $R_{\tau }=1$, and (**c**) $R_{\tau }=10$. N.B. *r_w_* = *r*/*w*, *T_max_* and *T_min_* are relative to $T_{00}=\frac{A(Ep)\cdot E_{p}}{\pi^{\frac{3}{2}}\rho C_{p}{w(E_{p,RR})}^{3}}$, which is the absolute maximum induced by one pulse and depends only on *Ep* in silica. Figure extracted from [40].

This relationship is illustrated clearly in Figure 1, extracted from [40].

When $R_{\tau }<1$, *T_min_* approaches *T_max_* regardless of the distance from the focal center. In this case, the stationary temperature distribution tends to follow a Lorentzian profile, characterized by a broad pedestal. Under this condition, using a mean temperature, which has a simpler analytical expression, is appropriate. Conversely, for $R_{\tau }>7$, the temperature distribution is closer to a Gaussian shape, exhibiting a narrow pedestal. It is interesting to note that for intermediate values of $R_{\tau }$, temperature oscillations can be neglected at a relative distance *r_w_* = *r*/*w* greater than 2, as shown in Figure 1. Considering these previous observations, we can generalize that the oscillations are negligible for *r_w_* = *r*/*w* > 2, regardless of the value of $R_{\tau }$. Therefore, the mean temperature can be expressed as $\frac{\sqrt{\pi }}{R\tau .\;r_{w}}\mathrm{erf}\left(r_{w}\right)$ (see Equation (A5)).

### 2.1. The Regime of Low Repetition Rate

The low repetition rate regime shows the advantages of minimizing thermal collateral damage and the heat-affected zone [42]. Consequently, ultrafast laser direct writing (ULDW) is widely regarded as an effective technique for inducing highly localized modifications and fabricating optical structures within/near the focal volume of various transparent solids [17,27,43,44,45,46]. In this non-thermal ULDW regime, where the repetition rate (*RR*) is typically in the order of a few kilohertz, the overall fabrication efficiency is limited by the relatively low pulse *RR*.

### 2.2. The Regime of High Repetition Rate

In contrast to the low *RR* regime, heat diffusion at high *RR* can extend thermal effects beyond the focal volume over longer time scales. This regime, referred to as thermal ULDW, is characterized by more extensive heat-affected regions. However, the size of this region does not increase significantly if the rise in *T*_00_/*R_τ_* is counterbalanced by appropriate control of the pulse energy.

While non-thermal ULDW has found widespread applications, localized thermal accumulation plays an important role in the ULDW by enabling the formation of diverse structural modifications in transparent solids and enhancing the performance of fabricated devices. For example, thermal accumulation can lead to a higher symmetry of waveguide cross-section, reduce propagation loss by self-annealing, and increase the fabrication efficiency [47,48,49]. Moreover, it can induce elemental redistribution and local crystallization, which are nearly unachievable in the non-thermal ULDW [27,50,51,52,53,54]. In thermal ULDW regime, the temperature gradient can act as a driving force to redistribute the elements of the material or reorganize the structures within the heat-affected zone [27].

## 3. Comparison of Thermal Treatment Curve with Transformation/Stability Curves According to the Mechanism

To demonstrate the practical significance of the aforementioned calculations, below, we discuss several problems where these equations can be applied to analyze temperature effects.

### 3.1. Type I

The refractive index at the origin of the type I is based on several contributions: the formation of point defects (molecular-level change, [55]), and a change in fictive temperature [56], equivalent to a change in the medium-range order of the glass that induces a local density change [55], which itself induces a stress–strain field (non-local).

The temperature dependence of these contributions are not the same. The thermal stability of the point defects is monitored by an activation energy distribution of VAREPA type ([39], studied at the end of this paper). The stress–strain field is initially proportional to the inhomogeneity of the density change produced by the irradiation. However, because it is non-local and the viscosity may be sufficiently low, another deformation field from the surrounding medium can screen the original deformation caused by the inhomogeneities. This viscosity-based deformation is governed by the relaxation time (*η*(*T*)/*G*, where *η* is viscosity and *G* is the shear modulus), similar to the fictive temperature *T_f_* responsible for the density change. Therefore, it is not possible to isolate the density-change-related index contribution without additional laser treatment over larger spatial and temporal scales. Consequently, the density change cannot be erased during the writing time in type I modification as the fictive temperature change can. The objective, then, is to achieve the greatest possible change in *T_f_*.

**Figure 2:**
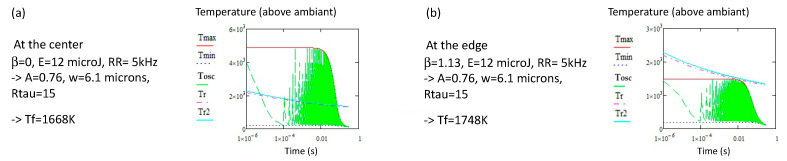
Comparison of *Tmin* (blue dashed line), *Tmax* (red) and *Tosc* (green dashed line, see Appendix B for its expression) for *RR* = 5 kHz. (**a**) at the center of the beam, (**b**) at the edge of the heat affected region. We note that *Tf* is changing a bit with the radius (slightly larger at the edge of the modified region). Note also that *Tmin* remains clearly below the relaxation curve for this *RR*. N.B. the relaxation temperature *Tr* and *Tr*2 have been shifted by 300 K.

**Figure 3:**
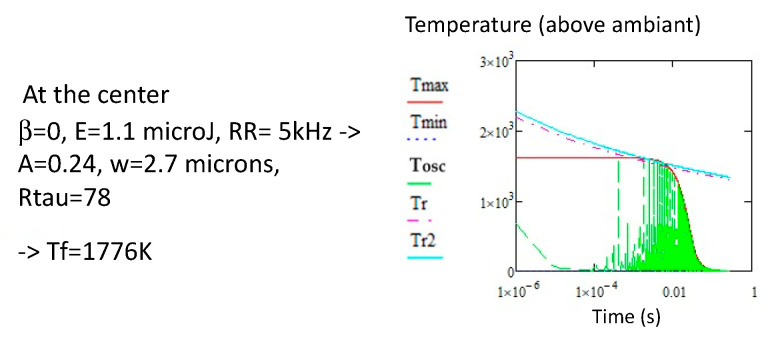
Computation with *Ep* = 1.1 µJ with the same *RR*. By comparison with Figure 2, we deduce the variation of *T_f_* with *Ep*; here, about −110 K for 11 µJ increase at 5 kHz. N.B. the relaxation temperature *Tr* and *Tr*2 have been shifted by 300 K.

**Figure 4:**
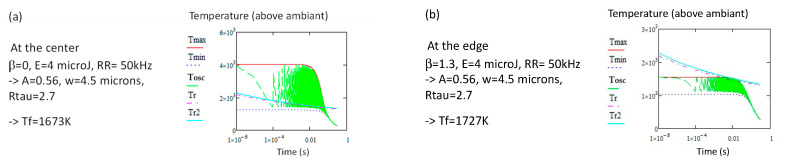
Computation with *RR* = 50 kHz. (**a**) at the center of the beam, (**b**) at the edge of the heat affected region. As for 5 kHz (Figure 2), *T_f_* is slightly increasing at the edge of the modified region. Note that the width of the heat-affected region is 30% larger than the beam. N.B. the relaxation temperatures *Tr* and *Tr*2 have been shifted by 300 K.

**Figure 5:**
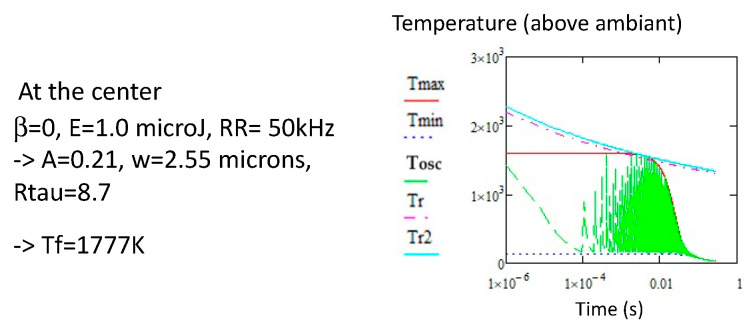
By comparison with Figure 4a, we see the variation in *T_f_* with *Ep*; a decrease of 100 K for an increase in Ep of 3 µJ. N.B. the relaxation temperature *Tr* and *Tr*2 have been shifted by 300 K.

**Figure 6:**
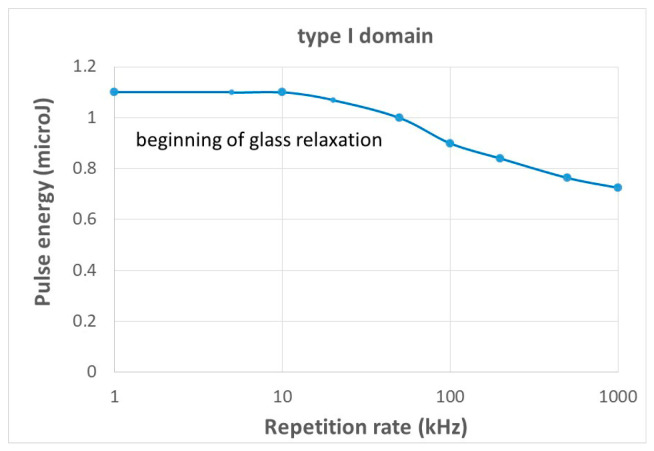
Beginning of glass relaxation curve (*T_max_* is overcoming the relaxation time).

This quantity, defined by Tool [57], depends on the thermal treatment according to the following equation: $\frac{dT_{f}}{dt}=\frac{T(r_{wd},t)-T_{f}}{\tau \left({T\left(r_{wd},t\right),T}_{f}\right)}$, where *T*($r_{wd}$,*t*) is the local thermal treatment curve, and $\tau \;\left(T\left(r_{wd},t\right),T_{f}\right)$ is the relaxation time *η* ($\;T\left(r_{wd},t\right),T_{f}$)/*G*, which is dependent on both the local temperature and prior thermal history that was previously established, *T_f_*, when the glass was out of thermal equilibrium [58]. Although there can be an effect of fictive pressure change [15] due to rapid thermal/Coulomb expansion, we neglect internal pressure changes for the sake of clarity and only consider the external temperature. We see from the equation that *T_f_* changes and follows the actual temperature only when the relaxation time is sufficiently short. During the cooling period, as the relaxation time increases, *T_f_* stops evolving. Therefore, computing *T_f_* requires prior knowledge of the thermal curve during ULDW treatment, for which an analytical expression of the temperature curve is available in [40]. However, the temperature oscillates between *T_max_* and *T_min_* during each pulse period, which complicates the direct solution of the differential equation. Therefore, instead of solving it explicitly, we compare *T*($r_{wd}$,*t*) with the local relaxation temperature. The relaxation fraction *x_R_* is classically defined as $x_{R}=1-exp(-\frac{t}{\tau \left(T\right)})$. For *t* = *τ* or 2*τ*, this corresponds to relaxation fractions of approximately 63 or 87%, respectively. By inverting this relation *t* = *τ* or 2*τ* = *η* (T)/*G* or 2*η* (T)/*G*, we obtain *T_r_* or *T_r2_*, representing the temperature corresponding to a given relaxation time. These values are plotted in Figure 2, Figure 3, Figure 4, Figure 5 and Figure 6 and are found to be closely aligned. Let us compare now this relaxation temperature with the treatment curve at any point of the material. Given a specific relaxation time *τ*, for t = *τ* or =2*τ*, if *T*($r_{wd}$,*t*) > *T_r_*(*τ*) *or Tr*_2_(*τ*) then *T_f_* = *T*($r_{wd}$,*t*). Conversely, if *T*($r_{wd}$,*t*) < *T_r_*(*τ*) or *Tr*_2_(*τ*), *T_f_* is frozen at the value *T_f_* = *T_r_*(*τ*) or *Tr*_2_(*τ*). Since *T_r_* and *Tr*_2_ are decreasing functions of *τ*, if *T*($r_{wd},$*t*) > *T_f_* for t < *τ*, then upon cooling, *T_f_* = *T_r_*(*τ*) or *Tr*_2_(*τ*). This is the last value of the fictive temperature and stands for a given β. One difficulty lies in estimating the total treatment duration before determining the final value of *T_f_*. Owing to the radial symmetry of *T*($r_{wd}$,*t*) around r = 0, the full treatment time is twice the scanning time from the center to a given radius. On the other hand, the temperature oscillates between *T_max_* and *T_min_*. Therefore, if *T_max_* is smaller than *T_r_* or *T_r2_*, *T_f_* will not change, regardless of the duration. This defines a threshold condition: a minimum temperature is required to induce an increase of *T_f_*. This is particularly relevant because the initial fictive temperature of the glass is typically much lower than the final one in the heat-affected region (ca. 500–700 °C against 1300–1500 °C), along with the corresponding density change [59] and refractive index [60]. In contrast, if *T_min_* exceeds *T_r_* or *T_r2_*, the change would be completed—this may occur under conditions of heat accumulation alone. Consequently, a significant refractive index change cannot be achieved at a high *Rτ* (i.e., low *RR*). This reasoning can be applied whatever the coordinates of the material point (α,β,γ). In such a way, as we know the thermal treatment curve for any point, we obtain the distribution of the fictive temperature according to β (or the center of the written line) for a given set of parameters v (the scanning speed), *Ep*, and *RR*. A few examples are shown for silica in Figure 3, Figure 4, Figure 5 and Figure 6, deducing the following.

Figure 2a show the evolution of the temperature of a point at the center of the line. Tmax crosses the *T_r2_* at 1668 K, defining the *T_f_* for a total efficiency. However, this is not the case, as *T_min_* is largely below *T_r2_* for the laser parameters used (12 µJ, 5 kHz, 100 µm/s). *T_f_* is therefore slightly larger.

Comparing Figure 2a,b leads us to see that *T_f_* has a tendency to increase at the edge of the heat-affected region. β = 0 means the center of the line, and β = 1.13 means that beyond 1.13 w, the *T_f_* will not change anymore and stay at the initial value. The same observation can be achieved from Figure 4a,b obtained from 4 µJ, 50 kHz, 100 µm/s, and thus with some heat accumulation.

Increasing *Ep* leads to a decrease in *T_f_* of a hundred of K, as shown in Figure 2a and Figure 3 for 5 kHz or Figure 4a and Figure 5 for 20 kHz. The variation depends on *Ep* (about −110 K for 11 µJ increase at 5 kHz or −100 K for 3 µJ at 50 kHz); this variation increases in *RR* due to heat accumulation.

Varying *Ep* and *RR*, we can also plot a limit below which the modification is not possible (see Figure 6). We see that the limit is weakly dependent on *RR* as it is experimentally observed [18]. However, the pulse energy values are slightly larger than the experimental ones (exp. ca. 0.5–0.7). We also note that the width of the modified region can be larger than w if the pulse energy is large enough (Figure 3, 1.13 w for 5 kHz or Figure 4, 1.3 w for 50 kHz), but the most sensitive parameter is the beam scanning speed. At increasing speeds, the width of the modified region is narrower if the pulse energy is not increased, but in this case, *T_f_* will be significantly smaller so the refractive index, especially at large *RR* (small *R_τ_*). At constant speed, *T_f_* is *E_p_*-dependent (−10 K/µJ at 5 kHz, or −33 K/µJ at 50 kHz). This is due to the fact that the pulse period becomes smaller than the thermal diffusion time; thus, there is no interest in increasing these parameters, except to widen the heat-affected zone. On the contrary, the most active parameter is the scanning speed. *T_f_* increases with it, and is roughly proportional to *ln*(*v*), as we can see in Figure 2, Figure 3, Figure 4 and Figure 5. Thus, both density change and refractive index also increase in silica [61] with the scanning speed.

### 3.2. Type II

In the case of pNG writing, the industrial objective is to write a large-form birefringence (retardance) as fast as possible. However, their amplitudes are dependent on lineic deposited energy density, i.e., proportional to *Ep.RR*/*v* or the pulse energy density (*Ep*.*RR*.2 *w*(*Ep*)/*v*) ([3,62,63]). Therefore, if v is increased, *Ep* or *RR* should be increased at the same time. In such a way, the thermal effect, which is mainly monitored by *Ep* and *RR*, increases and may destroy the previously written modifications (at least partially). It is thus necessary to have an approach for modeling the parameters. Therefore, we have to compare the thermal treatment applied during scanning with the stability curve of the pNG. This is defined from the RP model [36], and it is the stability of the nanopores at the base of type IIp.

#### 3.2.1. Thermal Stability of Type II (pNG)

**Figure 7:**
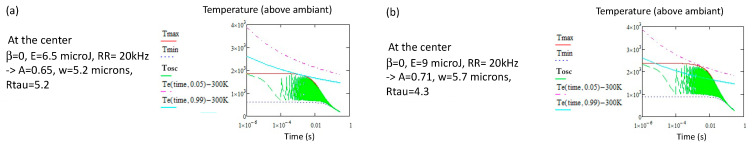
Erasure conditions for pNG at 20 kHz and *v* = 100 μ/s, at the center; (**a**) the solution for *Tmax* to reach the 5% erasure is 6.5 μJ, so with *w* = 5.2 μm, *A* = 0.65 and *Rτ* = 5. The time appears at 0.002 s. (**b**) The solution for *Tmin* to reach the 99% erasure is 9 μJ, so with *w* = 5.7 μm, *A* = 0.71, and *Rτ* = 4.3. The time appears at 0.004 s.

**Figure 8:**
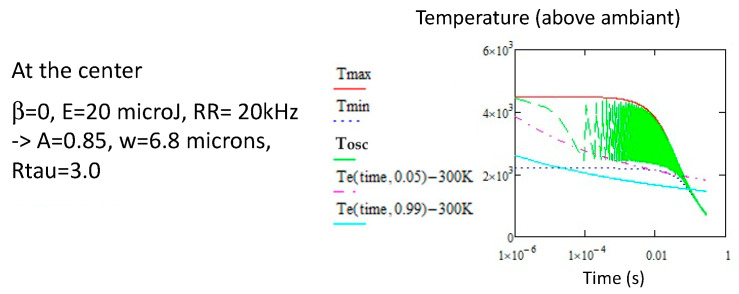
Conditions for complete erasure of pNG (Tmin overcomes the 99% stability curve) for 20 kHz, *v* = 100 μ/s, solution with 20 μJ, so with *w* = 6.8 μm, *A* = 0.85 and *Rτ* = 3. The erasure time appears at 0.01 s.

**Figure 9:**
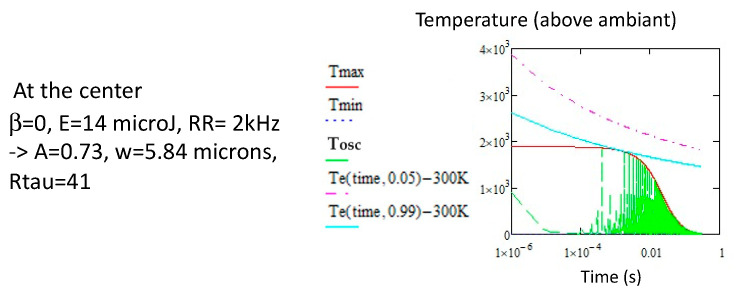
Erasure conditions for pNG at 2 kHz and *v* = 100 μ/s; the solution for *Tmax* to reach the 5% erasure is 14 μJ, so with *w* = 5.84 μm, *A* = 0.73 and *Rτ* = 41. The time appears at 0.002 s. Note that *Tmin* is no longer influenced by the pulse energy. Total erasure is never possible.

The erasure temperature of the pNG for, e.g., 30 min (T_30mn_ (pNG)) is demonstrated to be based on Rayleigh–Plesset (RP) equation [36], taking into account the erasure of nanopores that compose the pNG structure. The associated optical response, which is the birefringence normalized relative to its initial value before any thermal treatment (*B_norm_*), is proportional to *R_norm_*^6^. Here, *R_norm_* is the pore radius, and it is normalized with respect to its initial value (*R_ini_*) as for *B_norm_.* Consequently, it has been shown that one obtains the following expression for isothermal treatment:

(1) $$R_{norm}=1-\frac{\sigma .∆t}{2.\eta \left(T\right).R_{ini}}\Rightarrow \frac{\sigma .∆t}{2.\left(1-B_{norm}^{\frac{1}{6}}\right).R_{ini}}=\eta \left(T\right)\Leftrightarrow T\left(B_{norm},∆t\right)=T_{0}+\frac{B}{-A+log\left[\frac{\sigma .∆t}{2.\left(1-B_{norm}^{\frac{1}{6}}\right).R_{ini}}\right]}$$

where *σ* = 0.3 J/m^2^ (surface tension); *R_ini_* = 70 nm, we can define the temperature limit according to Δ*t* with *B_norm_* = 5% and 99%; and η is the glass viscosity (*A*, *B*, and *T*_0_ are fitting coefficients reported in Appendix A). The corresponding curves are reported in Figure 7, Figure 8 and Figure 9 in the next section (blue and red dashed lines, respectively). The corresponding temperature is noted as *Te* and corresponds to isothermal treatment.

#### 3.2.2. Limitation of the Processing Window of Type II

We intend to establish an *E_p_-RR* landscape for the pNG, i.e., what can be the pulse energies accessible according to the repetition rate [34] (see Figure 2 in Xie et al.’s study). For that purpose, as we know, the erasure temperature (*T_e_*) of such a structure as a function of annealing time using Equation (1). Now, this temperature limit can be compared to the given thermal treatment the material undergoes during laser irradiation when it is no longer irradiated. This means that when a point in the material, after having been irradiated, leaves the beam, it then experiences only the temperature distribution. For that, we have considered that the time of in situ annealing during writing begins when a given point of the material exits from the beam, so when *r_wd_* begins to be larger than w after crossing the beam.

This defines the origin of time of the treatment. There are three related curves for each given value of *β*. For the figure, we have considered β = 0 (the center of the line). The temperature oscillations are in green. They are located between *T_max_* and *T_min_*, the red and blue dashed curves, respectively.

As we can see, with the laser parameters we have chosen (6 microJ, 20 kHz, Figure 7), the temperature oscillations at the center progress below the erasure temperature until they approach the curves *Te* and possibly cross them. It is not exactly an isothermal process, and this decreases the efficiency of the treatment. Note that for *R*τ small (small oscillations) or for a radius distance larger than 2 w, a mean temperature can be used, simplifying the analysis. Of course, *T_max_* and *T_min_* only decrease as the point leaves the beam and we look for its survival. This approach is similar to the one for type I, but for different finality.

There are three particular points to allow for precision.

We note that it is possible that *T_max_* only touches the annealing curve corresponding to 5% erasure of the nanopores (Figure 9 above). In such a way, we obtain a couple (*Ep*, *RR*) of the limit below which pNG is not significantly erased, even partially. This leads to plot the blue curve in Figure 7. If this curve is overcome, the pNG will be partially destroyed and *T_max_* may meet the stability curve for 99% erasure (Figure 7 below). This does not mean that pNG will be completely erased, as a part of the oscillations are below this curve. It is just an intermediate destruction of the pNG (this curve is shown in Figure 7, the red dashed curve). For a complete erasure of the gratings, *T_min_* needs to overcome *Te* (99%). This is shown in Figure 9 by the blue dashed curve. The striking feature is that it needs a quite strong increase in *E_p_* for low *RR*, and we can even say that at a low repetition rate, the written pNG cannot be totally erased. Indeed, for total erasure during writing, when *T_min_* is close to zero (Figure 8), the oscillations are so large that the annealing will never be efficient. Finally, the curve for 5% compares rather well in shape with the experiment [34].

The limit at low *RR* becomes independent of the *RR*. Then, decreasing at larger *RR* for showing that with heat accumulation, it is almost impossible not to erase the nanopores during the scanning, and even in the course of the irradiation by a fast scanning that preserves the minimum number of pulses for a significant retardance (at least 10 pulses [64]). A figure of 10 kHz appears to be a good compromise.

For comparison with experimental results, we have chosen the parameters in the experiment in [34]. They used pulses of 800 fs, and they wrote at 100 microns/s. In this, it is important to note that the maximum value for the pulse energy is at a low frequency and reaches about 12 microJ whereas it is published at 5 microJ.

**Figure 10:**
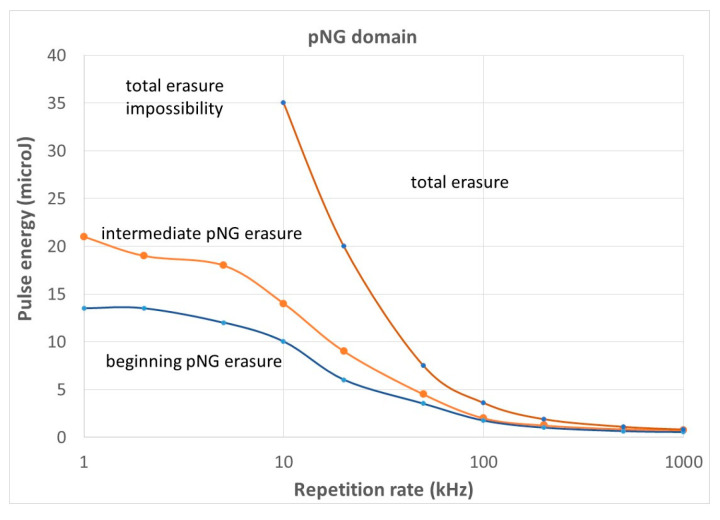
Landscape *Ep*-*RR* for type IIp according to the RP model and the thermal model. The curve at the lowest pulse energy is for the beginning of pNG erasure (*T_max_* is just touching the stability curve for 5% erasure). The curve at the highest pulse energy is for total pNG erasure (*T_min_* reaches the stability curve for 99% erasure). It is interrupted for *RR* lower than 10 kHz when pulse energy becomes too large to be applicable. In that case, it is considered that the total erasure is no longer possible. The curve for intermediate pNG erasure is for when *T_max_* reaches the stability curve for 99% erasure.

The shape of the curve in Figure 10 for the beginning of erasure (*T_max_* reaches the curve for 5% erasure) agrees with the experiment with a plateau at low *RR* and a decrease as long as *RR* is increased. However, there are some discrepancies: the value at the plateau is higher than in the experiment (14µJ instead of 5 microJ), and the RR at the inflection point is too low (20 kHz instead of ca. 200 kHz). The temperature is ruled by T_00_ at a low *RR* (no heat accumulation), $T_{00}=\frac{A(E_{p})\cdot E_{p}}{\pi^{\frac{3}{2}}\rho {\;C}_{p}{w\;(E_{p},RR)}^{3}}$. For a constant temperature of *Te* (5%), an excessively large *Ep* means an excessively large beam width in the model. On the other hand, the position of the inflection point is defined by the appearance of heat accumulation, i.e., at $R_{\tau }=7$ [40], since $R_{\tau }=\frac{4D_{th}}{RR\;{w(E_{p,RR})}^{2}}$, and an excessively small *RR* also means an excessively large beam width for the fixed $R_{\tau }$ considered here. On the other hand, a test with the model suggested that a reduction of about 40% is enough for explaining the discrepancy. This point will be discussed in the Section 4.

We also note that, for low *RR*, it is not possible for *Tmin* to overcome the 99% stability curve, and thus it is not possible to erase completely the nanograting. What is, thus, the limitation given in [34]? It is a level off of the retardance on pulse energy, i.e., the increase in the volume of NG is probably counterbalanced by a partial erasure.

Moreover, with the model we have used, the properties at the edge (*β* = 1) are the same as in the middle of the NG (*β* = 0). The erasure rate is the same.

Comparing the spatial extend of type I and type II, the last one is limited by the beam, so it has half the width of *w*, whereas for type I, the maximal radius is defined with the same laser conditions that lead to an excessively lower treatment curve for *T_f_* change (*β_max_*). When *β_max_* > *w*, it is possible to see a contrast in phase shift that overflows the birefringent area. *β_max_* can be larger or smaller than w, as they do not originate from the same mechanism. *w* is defined by direct optical effect, whereas *β_max_* is a thermal effect [65].

The landscape in Figure 10 allows us to explain that an increase in RR with constant *E_p_* leads to a decrease in the size of the porosity and in the number of nanoplans’ coalescence [66].

### 3.3. Type III, the Same Approach as Above (Comparison of Stability and Thermal Treatmentl Curve)

The modification called type III is mainly a hole or nanobubble. The formation mechanism can be decomposed into four stages. Stage 1 is the energy deposition when the hole is not yet formed (electron excitation, etc.). Basically, it is the same problem than the previous type, except that the pulse energy is slightly larger. There is ionization and weakening of the chemical bonding within a radius *w*1. In Stage 2, there is local thermalization in a couple of ps; the local temperature increases and the matter expands on the effects of the phonon population and Coulombian repulsion [10] in a few ns [67]. The beam radius *w*1 increases suddenly until *w*2. In Stage 3, the phase transformation occurs and the bubble forms after a time corresponding to the stress propagation, also evaluated to a few ns. A densified shell surrounds the bubble due to matter conservation. A part of the deposited energy is used in this transformation. *w*2 evolves into *w*3. In Stage 4 (final), if the temperature around the bubble is large enough, the hole can decrease in size until disappearance, totally or partially depending on the laser parameters used, the gaz contained in the bubbles (internal pressure), and its size. This is the definition of a limitation for type III if a well-defined hole is necessary for the application.

Stage 4 can be thus modeled on the basis of *w*3, and the matter dynamics is driven by the RP equation, as we have seen above with type II. The initial radius alone is much larger (two-order larger) and thus the stability is higher than those in Figure 7, Figure 8 and Figure 9, and thus may lead to a higher maximum pulse energy. Above this energy limit, the hole may collapse, but the nanobubble size being not negligible in front of w may modify the problem of homogeneous absorption.

Another limitation appears in this scheme: for lower pulse energy, the phase transformation may not be achieved, and therefore there may not be a hole. This is a low-energy limitation of type III.

### 3.4. On VAREPA Systems

A system that follows the VAREPA approach [39] has its thermal stability that can be described by a master curve (the relevant normalized quantity like the Bragg diffraction efficiency or the retardance of UV-induced Bragg gratings or points defects), i.e., time and temperature can be gather in a unique variable called demarcation energy (*Ed*) with the most frequent expression *Ed* = *k_b_Tln* (*k*_0_*t*) [39]. The master curve can be written as MC (Ed). On this, one can be decided that any thermal treatment should not erase more than a fraction of the related quantity to *MC* (*Ed*). To this limit corresponds a demarcation energy limit that one not to overcome. Let us call this the last *Ed_limit_*, which leads to *T* = *Ed_limit_*/*k_b_ln* (*k*_0_*t*) that can be considered as the stability curve.

Now, consider our thermal treatment during writing (*T_max_* (*r_wd_*,*t*) and *T_min_* (*r_wd_*,*t*)) with a relevant time origin *t*_0_ from the active period, like it was for description of the other types. By comparison of the two curves, like it has been performed in Figure 6, Figure 7, Figure 8 and Figure 9, we can deduce a landscape such as that in Figure 10 for the related modification.

## 4. Conclusions

In this paper, we have considered the thermal dependence of several modifications induced by an fs laser. We have compared the thermal treatment curve at any point of the material that crosses the beam (using previous simple analytical description) with the transformation/stability curve of modifications. In such a way, we have shown the effect of laser parameters.

For type I, it needs to maximize the fictive temperature as the phase shift or index change vary from this quantity. *T_f_* dependence on *RR* is weak without heat accumulation. It decreases with *Ep*; therefore, there is no interest in this direction. The most sensitive parameter is the scanning speed, which increases the cooling. *T_f_* rules approximately with *ln*(v).

For type II, its total erasure is not possible without heat accumulation. Therefore, the largest retardance can be obtained at low *RR*, for which *R_τ_* > 7 and where *E_p_* can be maximized.

For type III, we only forecast that stability is larger than type II, so the erasure limit is higher than this last one, with the problem being the light absorption of an inhomogeneous structure.

The approach described in this paper is applicable to any other transformation, providing the thermal transformation/stability curve is known.

However, the values of the limits that we have obtained appear too large compared to the experiment by about 40%. This arises probably by a description of the beam distribution too simple (one Gaussian shape). We suggest that the effective beam is composed of at least two components: one narrower, from the optical beam, giving rise to thermal effect and another, much larger one, originating from the multiple scattering, which is therefore attenuated and gives rise to the NG organization. We can add also that a clamping effect of the excited electron density that limits the absorbed fraction of the pulse energy [68] has not been taken into account in the *A* (*E_p_*) coefficient and may improve the agreement. As the Fourier equation is linear, the superposition of the two Gaussian sources will lead to the superposition of the two solutions, but this is a refinement that needs additional experiments that are not available at the moment. These are the future directions for an improved, simple model.

## Figures and Tables

**Figure A1:**
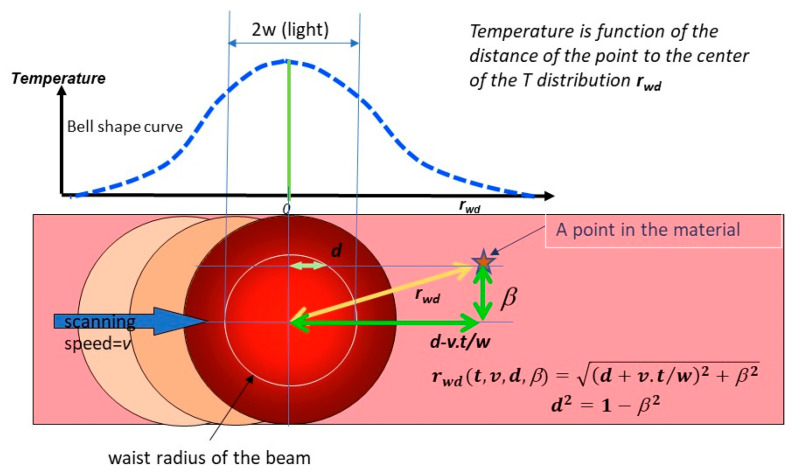
Scheme of the different variables used in the calculations. d is shown in the case of type II, for type I or III, d = 0.

## Data Availability

Data is contained within the article.

## Appendix A

**Table A1:** Glossary of laser and material parameters for silica.

Parameters	Definitions	Units
***T*** **_00_**	Temperature increment above room one produced by a single pulse	see equation below
***T_min_***	Minimal temperature of the temperature oscillation at the steady state	see equation below
***T_max_***	Maximal temperature of the temperature oscillation at the steady state	see equation below
***T_osc_***	Temperature oscillation at the steady state	°
***T_f_***	Fictive temperature (representative of medium-range order of the glass)	K
***T_r_* or *T_r_*** **_2_**	Relaxation temperature according to relaxation time for 63 or 87% of relaxed glass fraction (tool definition, see text)	K
***T*** **(** $r_{wd},$ ***t*** **)**	Temperature increment above room one at the distance $r_{wd}$,*t* and at time *t* This temperature can be either *T_max_*, *T_min_* or *T_osc_* according to the discussion.	°
***Te***	Temperature for erasing 5% or 99% of nanopores involved in type II modifications	K
		
$E_{p}$	Pulse energy	J
A	Fraction of absorbed light	none
		
$\tau_{RR}$	Period of the pulses	μs
f	Pulse repetition rate	MHz
w	Effective beam waist radius (at 1/e)	μm
		
κ	Thermal conductivity	1.09 W/(m.K) ^(1)^
ρ	Density	2200 kg/m^3^
$C_{p}$	Specific heat capacity	703 J/(kg.K)
$D_{T}$	Thermal diffusivity $D_{T}=\frac{\kappa }{\rho {.C}_{p}}$	7.06 × 10^−7^ m^2^/s
$\tau_{D}$	Heat diffusion time $\tau_{D}=\frac{w^{2}}{4D_{T}}$	μs
**η**	Viscosity ^(2)^	${log}_{10}(\eta \left(Pa.s\right))=-4.55+\frac{21254K}{T\left(K\right)-139K}$
		
Rτ	$\tau_{RR}/\tau_{D}$	none
		
ε	A small quantity of computational needs	none
**r_w_**	Normalized radius (r/w)	none
**r_wd_**	Normalized dynamic distance to the focus center (see Equation (A4))	none

(1) Using the empirical formula given in [69]. (2) From [70,71,72], Tg ≅ 1450 K, melting point around 1983 K.

## Appendix B

### Appendix B.1. Temperature Distribution Evolution During Laser Writing (Scanning Mode)

The formulae used in this paper were extracted from [40], and are recalled below. The numerical values of the required parameters are listed in Appendix A. It is also assumed that no endothermic reaction occurs in the dark that would consume a significant portion of the absorbed energy. The electronic band structure of the material and the non-linear absorption coefficient are assumed to remain unchanged during irradiation and considered temperature-independent. For better accuracy, the reference temperature should be chosen around the transformation temperatures of the relevant modification.

Consequently, the shape of the spatial temperature distribution is not time-dependent; the only time dependence is introduced by the beam scanning along the *α* direction and appears as *α* + *v.t*. The parameters *β* and γ are fixed, with the γ direction corresponding to the beam propagation axis. The origin of the coordinates is the focus center. The scanning speed (*v*) defines the number of pulses deposited immediately as *N_p_* = 2 *w.RR*/*v*. The laser pulse energy *E_p_* defines the maximum temperature increment *T*_00_, introduced by a single pulse above the initial temperature, as $T_{00}=\frac{A\cdot E_{p}}{\pi^{\frac{3}{2}}\rho C_{p}w^{3}}$, where *A* is the fraction of absorbed energy (previously defined); and *ρ* and *C_p_* are the glass density and heat capacity, respectively. Additionally, a normalized radius *r_w_* = *r*/*w* is defined, where r is the radial distance from the center of the beam focus (see Table A1).

The temperature oscillations occur between a maximum (*T_max_*) and a minimum (*T_min_*), which vary according to the laser irradiation conditions. After a sufficient number of pulses (*N_ss_*), both *T_max_* and *T_min_* reach a steady-state regime. Achieving this steady state is governed by the ratio *R_τ_* as previously defined, given by *R_τ_* = 4 *D_th_*/*RR w*^2^.

*N_SS_* corresponds to the minimum number of pulses necessary to reach the steady state. Its expression is given in Equation (A6) below. This calculation can be performed for either *T_min_* or *T_max_*. However, in this paper, it is specified that when *r_w_* > 2, the difference between *T_min_* and *T_max_* becomes negligible, as seen in [73]. Therefore, the conditions of *r_w_* and $R_{\tau }$ for neglecting the temperature oscillations are deduced, making the use of an average temperature *T_mean_* applicable (Equations (A7) and (A8)).

The static temperature distribution is mapped into the time domain to obtain thermal treatment curves. This is performed by replacing the static normalized radius $r_{w}$ by the coordinate $r_{wd}$, which represents the position of the point in the moving material, taking the beam center as a reference (see Figure A1). The relative position $r_{wd}$ in the beam is related to relative time by the following relation: $r_{wd}(t,v,d,\beta )=\sqrt{{\left(d+v.t/w\right)}^{2}+\beta^{2}}$. The time origin of the treatment for each modification is defined as the moment when the point of the material either experiences a temperature larger than the final fictive one for type I (due to the symmetry of the problem around the center of the focus, the time is twice the one from the focus center, i.e., d = 0, $r_{wd}(0,v,0,\beta )=\beta$) or when the point moves out of the beam, causing the temperature to significantly decrease (i.e., $r_{wd}(0,v,\sqrt{1-\beta^{2}},\beta )=1$ for type II or III).

While expressions are provided in this appendix, it is worth emphasizing that *T_max_* and *T_min_* are only function of parameters *R_τ_* and *T*_00_. In turn, $R_{\tau }=\frac{D_{th}}{RR{w(E_{p},RR)}^{2}}$ and $T_{00}\propto \frac{A(E_{p})\cdot E_{p}}{{w(E_{p},RR)}^{3}}$ are functions of *RR*, *Ep*, *A* and *w*. The latter two parameters are themselves functions of *Ep* and possibly of *RR*: *A* (*Ep*) and *w* (*Ep*,*RR*), as reported in [31], but they are not precisely defined. This issue has been previously discussed in [40].

### Appendix B.2. T_min_ and T_max_

The temperature induced by laser pulses will not increase indefinitely but converge to a finite value. This defines a steady state that corresponds to the equilibrium between the energy supplied by the laser and the energy diffusing out of the irradiated voxel.

From *N_ss_*_max_ together with the laser pulse *RR*, we know the time needed to reach the steady state. Accordingly, the time for reaching the steady state $\;t_{ss}^{0}$ is (considering the effective number to reach the *T_max_* limit) as follows:

(A1) $$t_{ss}^{0}=N_{ssmax}^{0}\tau_{p}=\tau_{d}\left[{\left(\frac{2}{R_{\tau }.\varepsilon .T_{max}\left(0,\infty \right)}\right)}^{2}-1\right]$$

(A2) $$\frac{T_{max}\left(r_{w},R_{\tau }\right)}{T_{00}}=\frac{\exp\left[-\frac{{\left(r_{w}\right)}^{2}}{1\;+\;x_{m}\cdot R_{\tau }}\right]}{{\left[1+x_{m}\cdot R_{\tau }\right]}^{\frac{3}{2}}}+\frac{\exp\left[-\frac{{\left(r_{w}\right)}^{2}}{1\;+\;{(1\;+\;x}_{m})\cdot R_{\tau }}\right]}{2{\left[1+\left(1+x_{m}\right)\cdot R_{\tau }\right]}^{\frac{3}{2}}}+\frac{\sqrt{\pi }}{R_{\tau }.r_{w}}erf\left[\frac{r_{w}}{\sqrt{\left[1+{(1+x}_{m})\cdot R_{\tau }\right]}}\right]$$

With:

*x_m_* = 0 if $r_{w}^{2}<1.5+\frac{2}{R_{\tau }}$ or x_m_ = 1 if $x_{m}=\frac{\sqrt{R_{\tau }}\sqrt{9R_{\tau }+32{\left(r_{w}\right)}^{2}}-3R_{\tau }-8}{8R_{\tau }}>1$,

$x_{m}=\frac{\sqrt{R_{\tau }}\sqrt{9R_{\tau }+32{\left(r_{w}\right)}^{2}}-3R_{\tau }-8}{8R_{\tau }}$ otherwise.

(A3) $$\frac{T_{min}\left(r_{w},R_{\tau }\right)}{T_{00}}=\frac{\exp\left[-\frac{{\left(r_{w}\right)}^{2}}{1+{(1+x}_{m})\cdot R_{\tau }}\right]}{2{\left[1+\left(1+x_{m}\right)\cdot R_{\tau }\right]}^{\frac{3}{2}}}+\frac{\sqrt{\pi }}{R_{\tau }.r_{w}}erf\left[\frac{r_{w}}{\sqrt{\left[1+R_{\tau }\right]}}\right]$$

With $R_{\tau }=\frac{4D_{th}}{RR{w(E_{p,RR})}^{2}}$, $D_{th}=\frac{\kappa }{\rho \cdot C_{P}}$ $T_{00}=\frac{A(E_{p})\cdot E_{p}}{\pi^{\frac{3}{2}}\rho {\;C}_{p}{\;w(E_{p},RR)}^{3}}$

In order to introduce the time (t) in the equations as the beam is progressively scanning and its distance to the point is changing, the *r_w_* previously provided is substituted by *r_wd_*, using the following relationship:

(A4) $$r_{wd}\left(t,v,d,\beta \right)=\sqrt{{\left(d+\frac{v\cdot t}{w}\right)}^{2}+\beta^{2}}$$

(A5) $$T_{osc}\left(r_{wd},{x_{bis},R}_{\tau }\right)\approx T_{00}\cdot \left(\frac{\exp\left[-\frac{{\left(r_{wd}\right)}^{2}}{1+x_{bis}.R_{\tau }}\right]}{{\left[1+x_{bis}.R_{\tau }\right]}^{\frac{3}{2}}}+\frac{\sqrt{\pi }}{R_{\tau }.r_{wd}}\left\{erf\left[\frac{r_{wd}}{\sqrt{\left[1+\left(1+x_{bis}\right).R_{\tau }\right]}}\right]-erf\left(\frac{r_{wd}}{\sqrt{1+R_{\tau }}}\right)\right\}\right)+T_{min}$$

With $x_{bis}$, the floating value of *t.RR* (that is, it takes a value from 0 to 1 along one temporal period).

Note that the time is introduced in the formula by $r_{wd}$ which is a “moving” $r_{w}$ (as the beam is scanning) and in x by rendering it periodic, i.e., in taking only the floating value of *t.RR*.

The corresponding signification of each term are schematically illustrated in the drawing Figure A1. Note that the d value sets the initial position of the beam along the writing axis and corresponds to the initial time t = 0 of the treatment. This one depends on the modification we are considering, but it is not awkward. For instance, for fictive temperature, initial time can be taken when the point is penetrating the beam, and for nanopores, it is when the point is living the beam (r_wd_ > 1) for crystallization it is when the temperature is overcoming the *T_ref_*. If a VAREPA approach is considered, the time origin has to be the same than in VAREPA framework, i.e., when the treatment begins to be active. On the other hand, since it is a function of *β*, an initial temperature reached by the position to set the initial treatment curve can be decided by solving *T_max_* (*r_wd_*(*t* = 0,*v*,*d*, *β*),*R_τ_*) = (*T_ref_* − *T_room_*)/*T*_00_.

The number of pulses for reaching the steady state and use the above formula is as follows:

(A6) $$N_{ssmax}^{r}>\frac{1}{R\tau }\left[{\left(\frac{2}{R_{\tau }.\varepsilon .T_{max}\left(r_{w},\infty \right)}\right)}^{2}-1\right]$$

When the mean T can be used, $\bar{T}\left(r,N\right)=\frac{1}{\tau_{RR}}\int_{\begin{array}{c} pulse\;period\\ at\;N \end{array}}T\left(r,t\right)dt$; the formula is even simpler to use after reaching the steady state. τ_RR_ is the period = 1/*RR*

(A7) $$\bar{T}\left(r_{w},R_{\tau },\infty \right)=\frac{\sqrt{\pi }}{R_{\tau }.r_{w}}\mathrm{erf}\left(r_{w}\right)$$

(A8) $$N_{ssm}^{r}\left(r_{w}\right)=\frac{1}{R_{\tau }}\left[{\left(\frac{2.r_{w}}{\sqrt{\pi }.\varepsilon .erf\left(r_{w}\right)}\right)}^{2}-1\right]$$

## Appendix C

The dependence of *A* (the absorbed fraction of pulse energy) and *w* (the half width of the beam at 1/e) with the laser parameters in our problem is already shown in [74]. It is shown that the theoretical computations are not able to predict these quantities with reliability, and thus only a comparison with experimental results allows us to estimate them. In particular, the information about *w* is from [74], considering that the birefringence width, arising mainly from NG, represents the beam width. These data are completed with measurements performed in our team on porous-based NG birefringence from other materials [11]. The results are provided in Figure A2. On *A* values, the solutions obtained herein lead to a lower fraction than silica or LNS glasses (see Figure A2. On *w*, this glass confirms what it was shown previously in [41] that the beam width is much larger than the optical width defined by the Kerr effect [75] and the broadening effect of excited electron density. It also clearly highlights a strong dependence of w on *RR* in this glass, along with a *E_p_*, more pronounced than the one of silica or LNS glass.

From the above compilation, and for the use of this paper restricted to silica, we have used the following fits:

For the absorbed energy fraction: *A* (*Ep*) = 0.171.*ln*(*Ep*(*μJ*) − 0.55*μJ*) + 0.345 for pulse duration around 800 fs. This fit applies for *Ep* > 0.7*μJ*.

For the effective beam radius (that seems not to be *RR*-dependent): *w* (*E_p_*,*RR*) = *w* (*E_p_*) = 1.43 μm *ln*(*E_p_*(μJ)) + 2.55 μm.

For these constraints, applicable for a pulse duration of 800 fs for *A* and in silica, *Ep* is limited to be above 0.7 microJ.
